# Author Correction: Concerted pulsatile and graded neural dynamics enables efficient chemotaxis in *C. elegans*

**DOI:** 10.1038/s41467-020-19316-5

**Published:** 2020-10-22

**Authors:** Eyal Itskovits, Rotem Ruach, Alexander Kazakov, Alon Zaslaver

**Affiliations:** 1 Department of Genetics, The Silberman Institute of Life Science, Edmond J. Safra Campus, the Hebrew University of Jerusalem, Jerusalem, Israel; 2 School of Computer Science and Engineering, the Hebrew University of Jerusalem, Jerusalem, Israel; 3 Edmond and Lily Safra Center for Brain Sciences, the Hebrew University of Jerusalem, Jerusalem, Israel

**Keywords:** Sensory processing, Systems analysis

Correction to: *Nature Communications* 10.1038/s41467-018-05151-2, published online 20 July 2018.

The original version of this Article omitted from the author list the third author Alexander Kazakov who is from the ‘Edmond and Lily Safra Center for Brain Sciences, The Hebrew University of Jerusalem, Israel’. The following was added to the Author contributions: ‘A.K. established the freely-behaving worm tracking system’. This has been corrected in both the PDF and HTML versions of the Article.

