# Supplementary material for: Concerted pulsatile and graded neural dynamics enables efficient chemotaxis in C. elegans
Source: Nat Commun. 2018 Jul 20;9:2866. doi: 10.1038/s41467-018-05151-2 (PMC6054637; doi:10.1038/s41467-018-05151-2)
Supplement: Supplementary file 1 — Supplementary Information [file 41467_2018_5151_MOESM1_ESM.pdf]

Supplementary Online Information

Concerted pulsatile and graded neural dynamics enables efficient chemotaxis in *C. elegans*

Itskovits et al.

Table of contents:

Supplementary notes

1. Microfluidic-based system for generating smooth gradients
   a. Modeling system dynamics for generating temporal gradients
   b. Practical constraints of the system
   c. Parameters used for generating the various gradient types

2. Chemotaxis simulations

Supplementary figures

Supplementary references

Supplementary notes

1. Microfluidic-based system for generating smooth gradients

a. Modeling system dynamics for generating temporal gradients

To generate each of the gradients we modelled the system dynamics, and analytically found the right parameters to be used for generating the chemical gradient of interest. The following section provides a detailed description of this model.

Let r(t) be the flow rate function of the syringe pump which carries the chemical of interest. We use a total constant flow rate R, meaning that the buffer syringe pump flow rate is R-r(t). We denote V to be the mixing chamber volume and F(t) to be the volume of odorant solution (in μL) inside the mixing chamber. The change of F(t) in time is given by the difference between the odorant entering the mixing chamber and the odorant exiting it:

$$(1) \quad \frac{dF(t)}{dt} = r(t) - R \cdot \frac{F(t)}{V}$$

Here r(t) is the odorant volume entering the mixing chamber every second, and $R \cdot \frac{F(t)}{V}$ is the amount leaving the chamber, as R is the total volume leaving the chamber and $\frac{F(t)}{V}$ is the fraction of the volume in the mixing chamber originating from the odorant syringe.

As we are interested to control the concentration of the odorant exiting the mixing chamber (which eventually enters the microfluidic device), we need to find the flow rate that will provide $C(t) = \frac{F(t)}{V}$. Hence, the pump rate should be:

$$(2) \quad r(t) = \frac{dF(t)}{dt} + R \cdot \frac{F(t)}{V} = V \cdot \frac{dC(t)}{dt} + R \cdot C(t)$$

This provides the flow rates needed for generating the desired function. For example, a linear temporal gradient is described by $C(t) = a \cdot t$ and therefore:

$$(3) \quad r(t) = V \cdot a + R \cdot a \cdot t$$

Our system parameters include V (the chamber size) and R, the constant flow rate to the chamber. These parameters dictate the maximal instantaneous change in concentration ($\frac{dC}{dt}$) that the system can support. We will derive the relationship between these parameters, and the maximal concentration change. The maximal flow $r(t)$ is bounded by R thus, from equation 2 :

$$(4) \quad \frac{dC(t)}{dt} \cdot V + C(t) \cdot R \leq R \Rightarrow \frac{dC(t)}{dt} \leq \frac{R(1 - C(t))}{V}$$

On the other hand, $r(t)$ cannot be negative:

$$(5) \quad \frac{dC(t)}{dt} \cdot V + C(t) \cdot R \geq 0 \Rightarrow \frac{dC(t)}{dt} \geq \frac{-R \cdot C(t)}{V}$$

Thus, to achieve a larger instantaneous concentration change rate, one can increase the constant flow rate or decrease the chamber's volume.

Moreover, it is clear from the derivation above that the upper and lower bound on $\frac{dc}{dt}$ are concentration dependent. If $C(t) \approx 1$(mixing chamber is almost full with odorant) then $\frac{dc}{dt}$ is bounded from above to be close to 0. And on the other hand, for $C(t) \approx 0$, $\frac{dc}{dt}$ is bounded from below by $\frac{-R \cdot C(t)}{V} \rightarrow 0$, which means the slope of the gradient has to become shallower as it approaches 0. Thus, as the gradient is approaching its maximal and minimal point, there are strict limitations on $\frac{dc}{dt}$. Due to these constraints, we avoided generating gradients that rise fast near the maximal concentration or drop fast near 0. For example, our sinusoidal gradient only reached a maximal point of 80% ($max(C) = 0.8$) from the maximal concentration in the syringe. In our experiments (**Figs. 1,3,4**), we used flow rates ranging between $R = 25\frac{\mu l}{sec}$ and $R = 150\frac{\mu l}{sec}$ and mixing chambers with volumes ranging between $V = 50\mu l$ and $V = 200\mu l$.

b. Practical constraints of the system

In addition to the theoretical constraints of the model, there are also practical constraints. In order for our mathematical description to hold, we assumed that the flow rate, set by the syringe pump, is the same flow rate to enter the mixer. For this to hold we had to avoid expansions due to flow pressure in the syringes or the tygon tubing that connects the syringes to the mixer. To allow this, we used a low resistance microfluidic chip (a wide and short tunnel), which requires low pressures to operate, and minimized tubing length from the syringes to the mixing chamber. Most importantly, we used glass syringes to reduce possible expansion due to the building pressure inside. Another requirement is that the fluid in the mixing chamber will have enough time to mix within it before it leaves towards the microfluidic chip. Assuming few turns of the magnetic stirring bar are enough to uniformly mix the fluids, this requires stirring at a rate of ~2

hz to allow smoothing of our minutes-long gradients. This rate was easily achieved using a standard commercial magnetic stirrer. Another point for consideration is the diffusion and turbulent processes during and along the flow inside the tube which may cause a small amount of the odorant to arrive prior to the expected timing based on calculations. This results in a neural response which may be observed ~1 minute ahead of its expected timing. An example of such a case is shown in Figure 1d.

c. Parameters used for generating the various gradient types

Throughout all experiments, except for the shallower linear gradient, the stimulus syringe contained the same diacetyl concentration (volumetric dilution of 10^{-4} diacetyl/buffer, equivalent to 1.15 mM).

Linear gradients. We used two linear gradients: one with a constant increasing slope of $20\frac{\mu M}{min}$ and a second, ten-times shallower, with a slope of $2\frac{\mu M}{min}$. For the shallower linear slope, the stimulus syringe contained diacetyl in concentration of 0.115 mM.

Exponential gradients. For the exponential gradients, we wished to avoid the abrupt and intense activation right at the beginning of the experiment which is associated with s the first drops of diacetyl that enter the mixing chamber. Therefore, we deliberately started by first generating a 10% step in the concentration followed by five minutes during which the neuron adapted to this low concentration. Only then, we started to exponentially increase the concentration for 13 minutes (**Supplementary movie 4**). Correlations based on the neural activity from these times are presented in **supplementary fig. 7**.

Hyperbolic tangent gradients. To generate a symmetric first-derivative sigmoid gradient, we used a hyperbolic tangent function. Four different types of gradients were used (**Fig. 3b**). For the longer gradient (**Fig. 3 biii**), we added a small increase in the concentration prior to the sigmoid start point to control for possible jump-start fluctuations in the system (similar to the exponential gradients above).

Sinusoidal gradients. Sinusoidal gradients were composed of four cycles, each lasted for five minutes. We set the basal concentration of the sine wave to 300 μM (**Fig. 4a, top**).

2. Chemotaxis simulations

Herein we provide the full description of the simulations, the wide range of parameters used, and the analytical solution for the case of linear gradients.

To contrast chemotaxis performance of the two strategies, we simulated animals navigating in two arenas: **(i)** an infinite 2D linear gradient, and **(ii)** a radial Gaussian gradient formed from a point source.

Both strategies rely on sensing the odorant concentration at each visited location and calculating the change in its concentration. We define C(t) to be the odorant concentration sensed at time t.

According to the classical biased random walk strategy, thought to be implemented by *E. coli* bacteria [1], the action in the next time point, t=T+1, depends solely on the sign of the first derivative currently sensed by the organism: $sign(\frac{dC}{dt}|_{t=T})$. The organism thus follows this simple set of rules:

1. If experienced a decrease in odor concentration in the last step (hence a negative derivative, $sign(\frac{dC}{dt}|_{t=T}) = -1$), then change direction with probability P_{-}.
2. If experienced an increase in odor concentration in the last step, (hence a positive derivative, $sign(\frac{dC}{dt}|_{t=T}) = 1$), change direction with probability P_{+}. Obviously, an advantageous strategy would be to require that $P_{-} > P_{+}$.

The decision is made only by $\frac{dC}{dt}|_{t=T}$, the current sensed derivative. In all cases, when changing direction, the new direction was chosen randomly from a uniform distribution in the range of [0, 2π].

To implement the second strategy where animals can also adapt to the first derivative, we modified the model to remember a set of previously sensed concentration derivatives $[\frac{dC}{dt}|_{t=T-M} \cdots \frac{dC}{dt}|_{t=T}]$, where M is the memory length. Importantly, this minimal addition to the previous model incorporates only a small set of parameters. This strategy follows this set of rules:

1. If $sign(\frac{dC}{dt}|_{t=T}) = -1$, change direction with probability P_{-}. This is the same rule used in the classical biased-random walk strategy.

2. If $sign(\frac{dC}{dt}|_{t=T}) = 1$, treat $\frac{dC}{dt}$ as a normal random variable and calculate the right sided Z-test p value of $\frac{dC}{dt}|_{t=T}$ compared to the derivative distribution of the last M steps: $[\frac{dC}{dt}|_{t=T-M} \cdots \frac{dC}{dt}|_{t=T}]$. The turning probability for this step is $P_{val} \cdot A$, where P_{val} is the p-value we have just calculated, and A is a positive constant (its relevance will become apparent below).

Similarly, the new turning direction is chosen randomly and uniformly in the range of [0, 2π].

Each of the above strategies can be described by the probability to turn when experiencing a positive first derivative (P_{+}) and by the probability to turn when experiencing a negative first derivative (P_{-}). In the case of classical biased random walk, the description is direct: we choose P_{-}^{1} and P_{+}^{1} prior to the simulation. In the case of the second strategy, ${P^2}_{+} = f(A)$, thus we can modify the value of parameter A to get any P_{+}. To correctly compare between the two strategies we compare them for the same values of P_{+} and P_{-}.

In practice, we chose P_{-}^{1} and P_{-}^{2} to be $P_{-}^{1} = P_{-}^{2} = P_{-}$. For each P_{+}, we chose $P_{+}^{1} = P_{+}$ and a value of A such that $P_{+}^{2} = f(A) = P_{+}$. To achieve this, following the simulation with a predetermined pair (P_{-}^{2}, A), we measured the number of turns performed while experiencing a positive gradient, and from this deduce the empirical P_{+} value. Note that by increasing A, we increase the probability to perform a turn. This allows using A values to match the mean probability P_{+} for a turn.

To quantify chemotaxis performance we used the widely-used estimate for chemotaxis efficiency, namely, the mean projection on the correct analytical maximal gradient direction [2]. For the classical biased-random walk strategy in a linear gradient, the mean projection is analytically solvable:

We define a linear gradient in the X direction of the form C(x,y) =Kx. In this case, the mean projection along the gradient is simply $\frac{X_n}{n}$, where X_n is the X position after n steps. For a random choice of a direction with a positive first derivative gradient, expectancy of the mean projection per step is therefore: E(+) = $\frac{1}{\pi}\int_{-\pi/2}^{\pi/2}\cos(\rho)d\rho = \frac{2}{\pi}$. Similarly, when moving down the gradient, the expectancy of the mean projection is: E(-) = $\frac{1}{\pi}\int_{\pi/2}^{-\pi/2}\cos(\rho)d\rho = \frac{-2}{\pi}$.

Thus, we know the expected mean projection when walking either up or down the gradient and are left to find the mean time spent in each state. The number of steps before turning in a positive gradient is a geometric random variable as each step has the same P_+ probability for performing a turn ($N_+ \sim Geo(P_+)$). Therefore, the expected number of steps when moving up the gradient is $E(N_+) = \frac{1}{P_+}$. Similarly, when moving down the gradient we find: $E(N_-) = \frac{1}{P_-}$.

So in total we get: $E(\frac{X_n}{n}) = \frac{2}{\pi} \cdot \frac{E(N_+)-E(N_-)}{n} = \frac{2}{\pi} \cdot \frac{\frac{1}{P_+}-\frac{1}{P_-}}{\frac{1}{P_+}+\frac{1}{P_-}} = \frac{2}{\pi} \cdot \frac{P_- - P_+}{P_- + P_+}$

This result indeed matches our simulation results (**Supplementary fig. 11**).

For other gradient conditions, simulations are needed to find the mean projections.

We first simulated the two strategies in an infinite linear gradient **(Supplementary fig. 11a-b).** The classical biased-random walk strategy behaves as intuitively expected and according to the analytical results **(Supplementary fig. 11c)**, where maximal scores are obtained for $P_+ = 0$ and $P_- = 1$; Namely, never turn when experiencing positive derivatives, and always make a turn otherwise. The worst performance is obtained along the $P_+ = P_-$ line, and crossing this line means that animals can no longer move up the gradient **(Supplementary fig. 11b-c)**.

In contrast, when simulating the second strategy (allowing adaptation to the first derivative, using a memory parameter M=30), we find that the maximal mean projection score is when $P_+ > 0$, meaning that it is beneficial to occasionally reorient even when moving up the gradient **(Supplementary fig. 11a)**. This may be surprising since according to our simulations, in each turn, the worm's new direction is chosen randomly from a uniform distribution, meaning that most chances are to turn into a less oriented trajectory. However, we find that for high values of P-, when the cost associated with a wrong turn is sufficiently small, taking these 'risks' may actually become beneficial **(Supplementary fig. 11a)**.

When comparing the scores of these two strategies, we find that adapting to the first derivative is always superior (or at most the same) to the classical biased-random walk strategy **(Supplementary fig. 11d)**. Importantly, we find this principle to hold for a wide range of P_+ and P_- values.

Note that the performance of the two strategies is identical only when $P_+ = 0$. This is because when $P_+ = 0$, the two strategies reduce to the same simple strategy: keep turning until experiencing a positive gradient and never turn again, yielding a mean projection score of $\frac{2}{\pi}$, as predicted. The ratio difference between the two strategies becomes larger around the $P_+ = P_-$ line. The reason is that around this line the classical biased random walk strategy fails to move the animal in the correct positive direction, whereas the first derivative adaptation strategy does **(Supplementary fig. 11a, 11b, black line)**. To estimate the difference in the performance of these strategies, we used the median of the fold improvement over all values of P_+ and P_-, as average fold improvement is infinite. Median fold improvement was found to be 30% (fold improvement is shown in **Fig. 5c** and **Supplementary fig. 11d**).

We next compared the two strategies in a 2D Gaussian gradient that may better resemble genuine ecologically relevant point-source gradients that animals encounter in nature. For simplicity, the gradient was kept constant during the entire simulation and did not evolve in time according to the diffusion equation. In this case, additional parameters need to be considered. We used the following parameters:

The gradient function was: $C(r) = e^{\frac{-r^2}{2\sigma}}$, with σ= 100 [au^2]

Speed: 1/simulation step [au]

Starting distance from source: 300 [au]

Stop distance: this parameter determines at what distance from the point source the simulation will stop: 30 [au].

Stop time: this parameter determines after how many time steps the simulation will stop if the stop distance was not yet reached: 3000 steps.

Memory length (M): 30 steps

Of note, the simulation results obtained in the Gaussian gradient are similar to those found in the linear gradient: the strategy to adapt to the first derivative outperforms classical biased

random walk strategy, and similarly, we find that the maximal mean projection score is when $P_+ > 0$ (**Fig. 5**).

We next repeated these simulations in the same Gaussian gradient while varying memory length (M). We found that increasing M improves chemotaxis directionality and this becomes particularly beneficial in regimes where P+ is close to P- . We also found that even a short memory length of M=2 steps is sufficient to greatly improve directionality compared to the classical biased random walk (**Supplementary Fig. 12**).

In order to check the robustness of the first adaptation strategy, we added noise to our simulations by adding a random noise perturbation at each simulation step. This perturbation was drawn from a Gaussian distribution with mean 0 and a constant standard deviation. We then repeated our simulations for increasing values of the standard deviation (**Supplementary Fig. 13**). We found that adding low levels of noise (which is small relative to the values of the mathematical gradient of the Gaussian at the starting point), has little effects on chemotaxis performance, while high noise levels greatly impair performance of both chemotaxis strategies. In all cases tested, adding noise does not change the fact that navigating with the ability to adapt to the first derivative is superior to the classical biased random walk strategy (**Supplementary fig. 13**).

Importantly, these simulations were not intended to fit *C. elegans*-specific chemotaxis parameters. Instead, we kept the simulations as general as possible with the sole purpose to contrast the two chemotaxis strategies, and by this, to extract the understanding for the superior performance of one strategy over the other. To provide a reliable comparison, we have systematically scanned a wide range of different values for the general motility parameters, such as turning probabilities (given a positive or negative gradient), memory times, and noise. Our results show that the adaptation to the first derivative strategy is superior to the classical biased random walk strategy across all aforementioned parameters. In addition, we simulated chemotaxis in a scale-free linear gradient, which allowed us to disregard specific diffusion parameters and to analytically solve chemotaxis performance.

In an effort to provide an intuitive understanding of the simulation parameters and their relatedness to the typical scales of *C. elegans*, one may consider the following: A typical chemotaxis assay places the worm ~ 5 cm away from the source[3]. In our simulations we used

300 steps as the distance of the virtual animal from the source, implying that each step is ⅙ mm ~ 200 um. Average worm speed is 0.2 mm/sec[4], thus each time step (*dt*) corresponds to ~1 second.

Supplementary figure 1

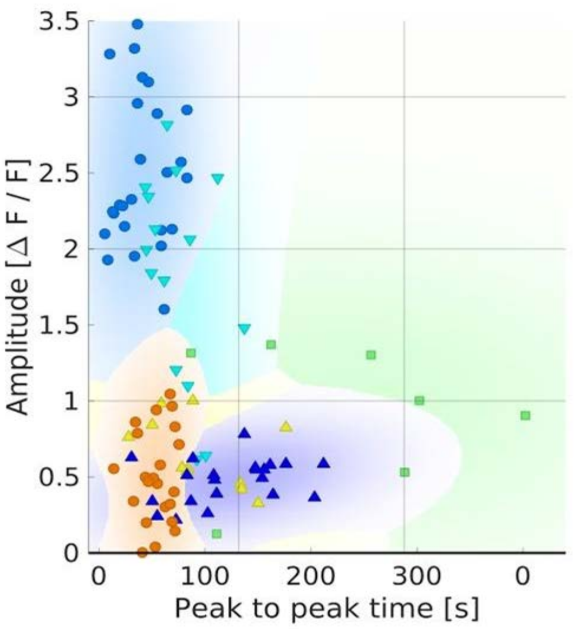

Supplementary figure 1| Worms exhibit individuality as each animal has its own pulsatile characteristics. Pulses are characterized by amplitude, decay time, and time interval between consecutive peaks. All pulses of the same six worms shown in Figure 1g are plotted in the amplitude and peak to peak time space. Pulses are color coded by the individual animal they were measured from. Pulses from the same worm are significantly more similar than pulses from different worms ($p \leq 10^{-6}$).

Supplementary figure 2

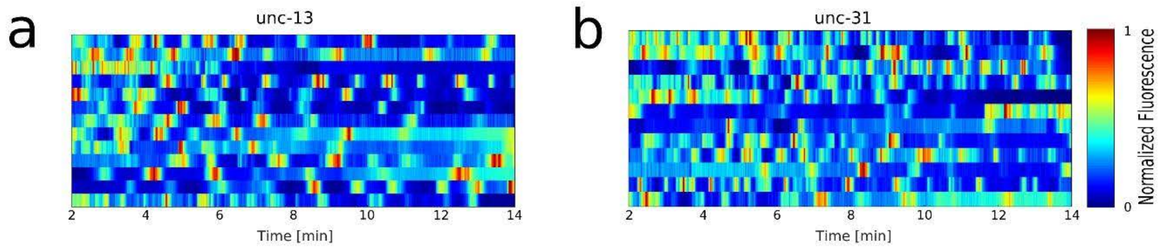

Supplementary figure 2 | Pulsatile activity is cell autonomous. Neural activity measured in animals defective in **(a)** neurotransmitter release (*unc-13(S69))*, and **(b)** neuropeptide secretion (*unc-31(e928)).* Pulsatile activity in response to increasing linear gradients is evident in animals defective in inter-neural communication suggesting that pulsatile activity is a cell autonomous process.

Supplementary figure 3

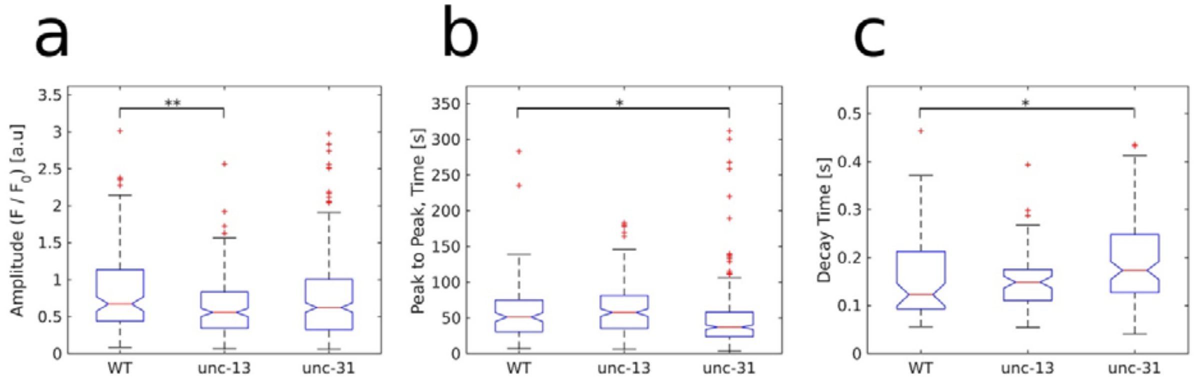

Supplementary figure 3 | Comparison of pulse characteristics between WT and transmission defective mutants. Comparison of amplitude **(a)**, peak-to-peak time **(b)** and decay time **(c)**, between animals defective in neurotransmitter release (*unc-13(S69), n=13 worms)*, neuropeptide secretion (*unc-31(e928), n= 13 worms)* and wild type animals (n=7 worms). Pulse amplitudes of *unc-13* mutant animals were significantly lower than those of the WT animals ($p<0.001$, Wilcoxon Rank Sum Test; number of pulses analyzed: n_{wt}=123, n_{unc-13}=199, n_{unc-31}=279). Compared to WT animals, *unc-31* mutants had a significantly lower peak-to-peak time ($p<0.03$, Wilcoxon Rank Sum Test; number of pulses analyzed: n_{wt}=123, n_{unc-13}=199, n_{unc-31}=279), and a significantly higher decay time ($p< 0.008$, Wilcoxon Rank Sum Test; number of pulses analyzed: n_{wt}=63, n_{unc-13}=68, n_{unc-31}=104). Blue box marks the 0.25 to 0.75 quantiles, and whiskers mark values between the 0.1 and 0.9 quantiles.

Supplementary figure 4

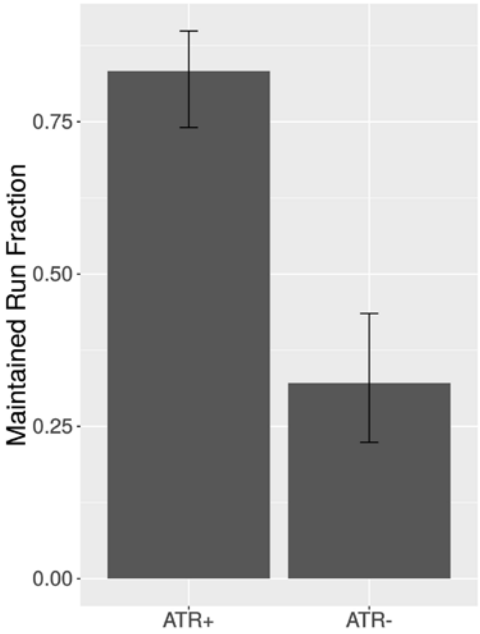

Supplementary figure 4| Worms maintain a run when AWA neurons are optogenetically activated. We used optogenetics to study the relationship between AWA activity and behavior. For this, we used worms expressing the light-activated channel, Chrimson, exclusively in AWA (CX16573**,** [5]). We placed a worm on an agar plate, when we visually inspected that it initiated a forward run, we turned on red light (545 nm) for 10 seconds. We then assayed whether it stopped the run during the 10 seconds that the light was on. Worms grown on ATR (a cofactor necessary for the optogenetic channel) were significantly more likely to maintain a run, indicating that AWA activity promotes forward movement (83% ATR+ as opposed to 32% ATR-, n_{ATR+}=96 trials on 32 animals; n_{ATR-} = 81 trials on 27 animals; $p < 10^{-10}$, X^2 test).

Supplementary figure 5

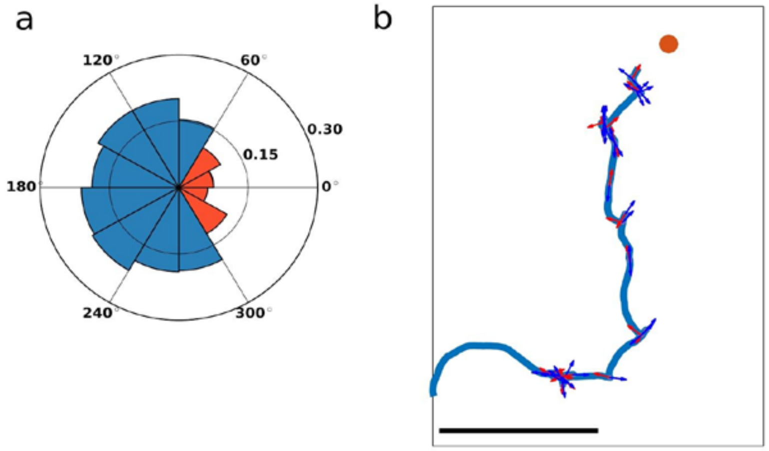

Supplementary figure 5| Worms perform turns even when sensing increasing chemical gradients (a) High-throughput chemotaxis assays demonstrate that animals will make turns even if initially directed towards the target. The angular statistics quantifies the probability of an animal to make a turn depending on its directionality before the turn. For example, worms crawling in a direction 60 degrees to the target have a probability of 0.1/sec to make a turn. **(b)** An exemplary worm trajectory during chemotaxis. The worm performs bouts of turning events (pirouettes) even when it moves directly towards the target, and hence sensing only increasing concentrations of the attractant. These results are compiled and calculated based on our previously reported large-scale chemotaxis assays[3]. Scale bar = 1 cm.

Supplementary figure 6

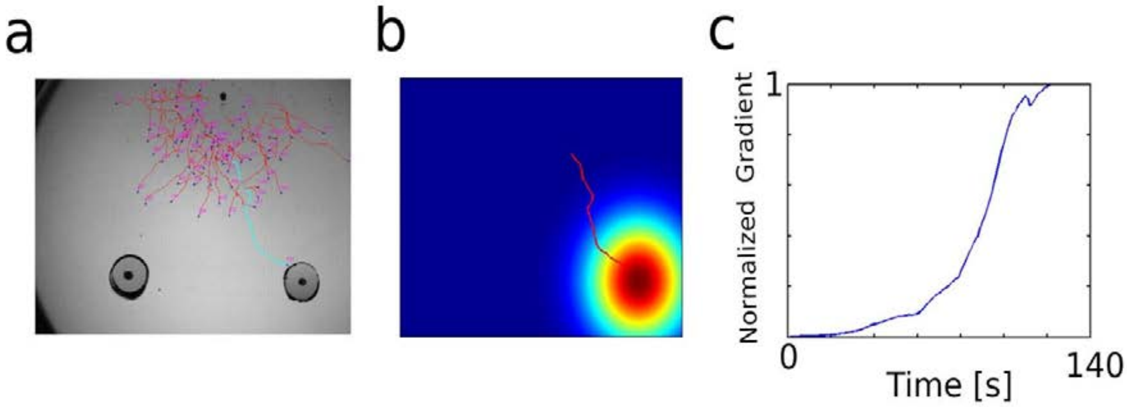

Supplementary figure 6 | Worms sense smooth Gaussian-shaped gradients when navigating towards a chemoattractant. Naturally-formed point source gradients are typically Gaussian (dictated by diffusion processes), and animals therefore face varying first derivatives of the chemical cue when navigating towards them. **(a)** A typical trajectory (cyan) of a worm navigating towards the chemical source. (**b)** A chemical source forms a Gaussian distribution (on a 2D plane) as governed by diffusion processes. **(c)** During chemotactic navigation in a Gaussian-type gradient, worms sense over time a stimulus that resembles a sigmoid function. This blue curve shows the concentration sensed by the cyan trajectory in (a) over time, given the concentration distribution in (b).

Supplementary figure 7

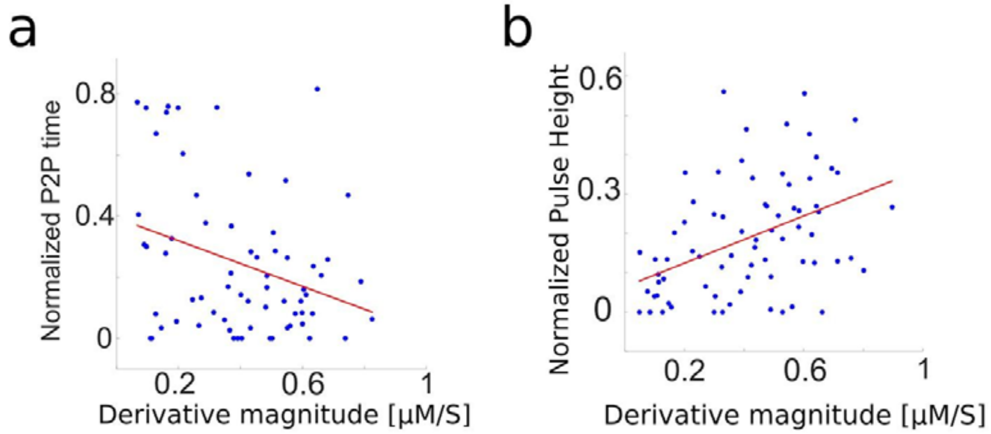

Supplementary figure 7 | The higher the magnitude of the first derivative, the higher is the amplitude and the frequency of the pulses. Peak-to-peak (P2P) times **(a)**, and amplitudes **(b)** correlate with the magnitude of the first derivative of the gradient. Data extracted from nine worms during an exponential gradient with temporally increasing first derivatives. Each blue dot marks a single pulse. Red line is the linear regression fit. Correlations are: **(a)** $\rho = -0.3$, ($p=0.01$, N = 75 pulses); **(b)** $\rho = 0.43$ ($p<10^{-4}$, N=75 pulses).

Supplementary figure 8

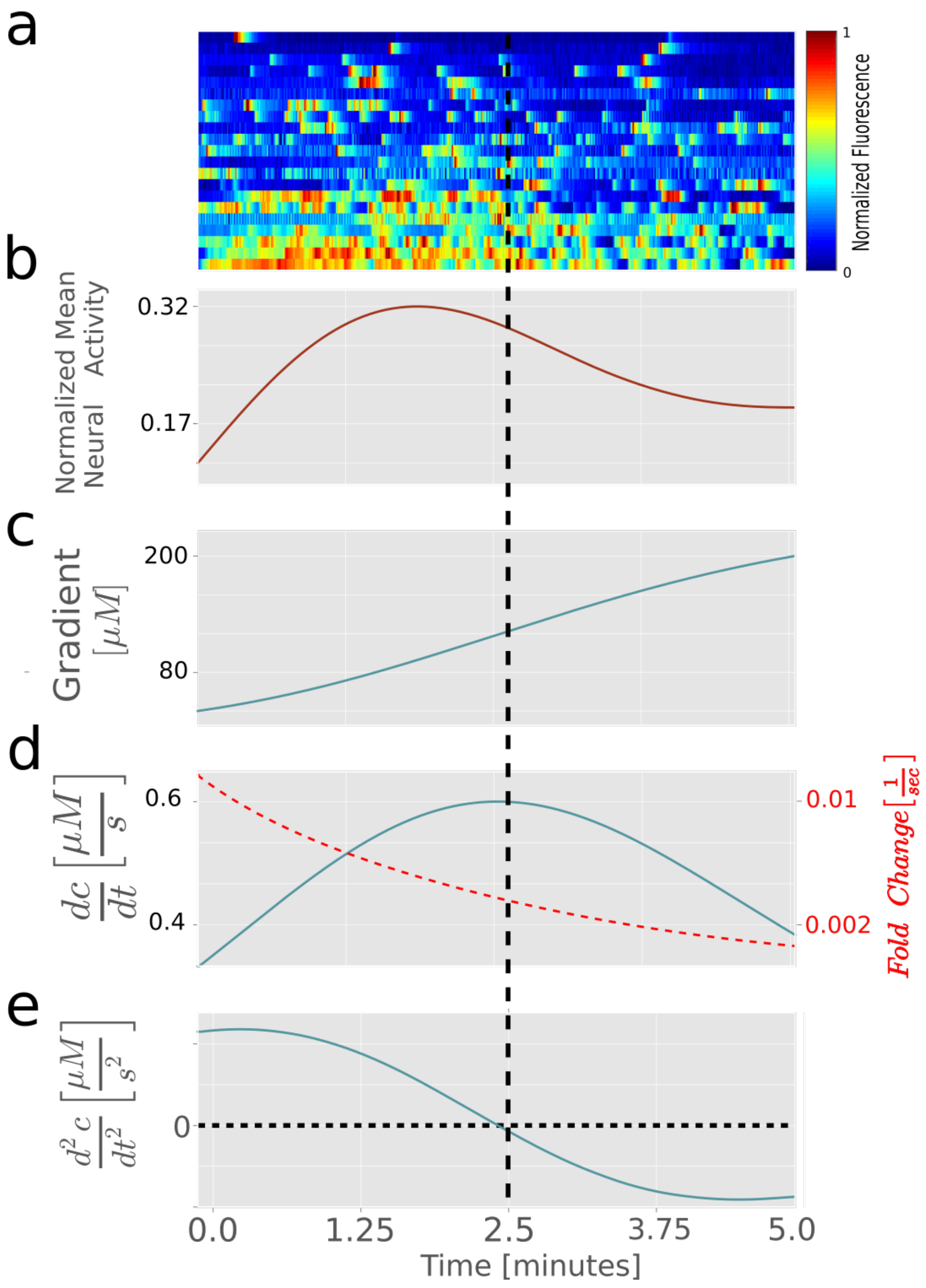

Supplementary figure 8 | Pulsatile activity better correlates with adaptation to the first derivative (and possibly with the 2nd derivative), rather than with the fold-change of the concentration. When presenting the worm with a sigmoidal chemical gradient, AWA pulsatile

activity **(a-c)** is high as long as the first derivative is increasing (positive second derivative, d and e). As the first derivative starts to flatten (although still positive), and the second derivative reaches zero, neural activity starts to decrease. Note, that the first derivative is symmetric around the dashed black vertical line. The fold-change of the concentration ($\frac{(\frac{dc}{dt})}{c}$ **, red dashed curve in (d)**) is constantly decreasing throughout the experiment. Thus, while fold-change responses may explain step-like functions [5], they are not likely to explain the pulsatile activity observed in response to smooth increasing gradients. Adaptation to the magnitude of the first derivative of the gradien**t** better explains the observed activity **(d,e blue curve).**

Supplementary figure 9

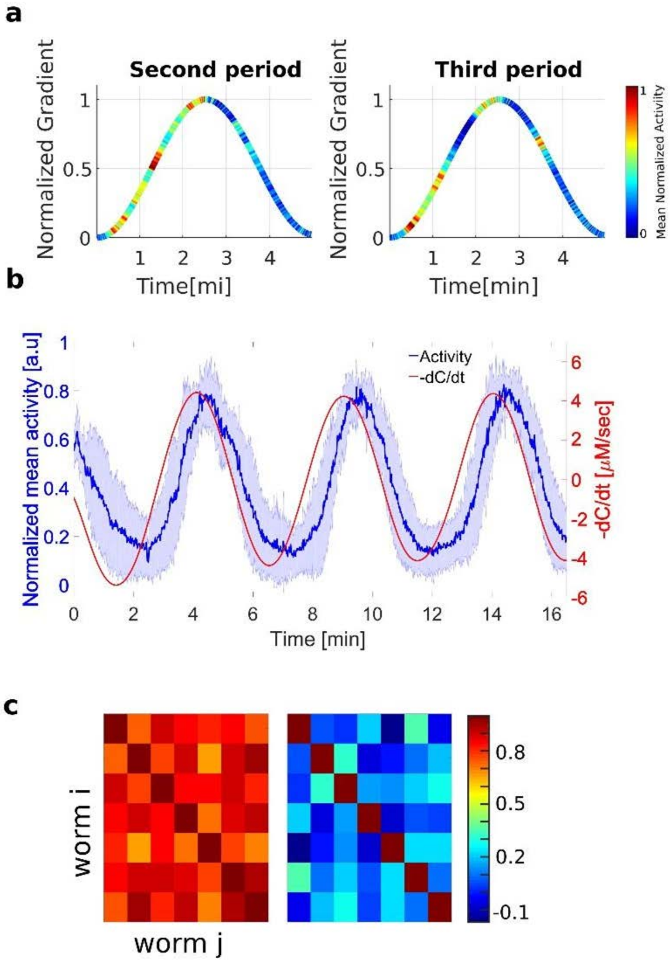

Supplementary figure 9 | AWA and AWC sensory neurons differ in their dynamics in response to smooth sinusoidal gradients. (a) Shown are the mean activities of AWA neurons for the second and third periods of the sinusoidal gradient (of the same neurons shown in Fig. 4c). Adaptation to the first derivative predicts that AWA activity will be lower during the third period when compared to the activity during the second period. However, we found that this activity is comparable and not lower, ($p > 0.05$). This raises the intriguing possibility that AWA neurons are actually sensitive to the second derivative of the gradient, as the activity decreased with the second derivative (Fig. 3) **(b)** Mean AWC activity (blue curve; shaded blue is activity standard deviation) and the first derivative of the gradient (multiplied by -1, red). It is evident that AWC activity follows the first derivative of the gradient with a lag time of 30 seconds. **(c)** Correlations in the neural activity among the different AWC (left panel), or AWA (right panel)

neurons, as observed in seven different worms in response to a sinusoidal gradient. Correlations were calculated based on neural activity during the 2nd, 3rd, and 4th periods of the sinusoidal gradient. In cases where two AWA neurons were recorded in the same worm, the neuron that showed a stronger mean activity was chosen for the analysis. While AWC activity is well correlated and therefore consistent between worms, AWA activity is poorly correlated. This, again, demonstrates the large variability in neural responses between worms (AWA correlation=0.11+/- 0.1, AWC correlation=0.83 +/- 0.1, $p<10^{-20}$, Wilcoxon ranked sum test was used to find that AWC paired correlations are significantly higher than those of AWAs, $p<10^{-7}$). Data shown in this figure is taken from the activity plots in figure 4a-c.

Supplementary figure 10

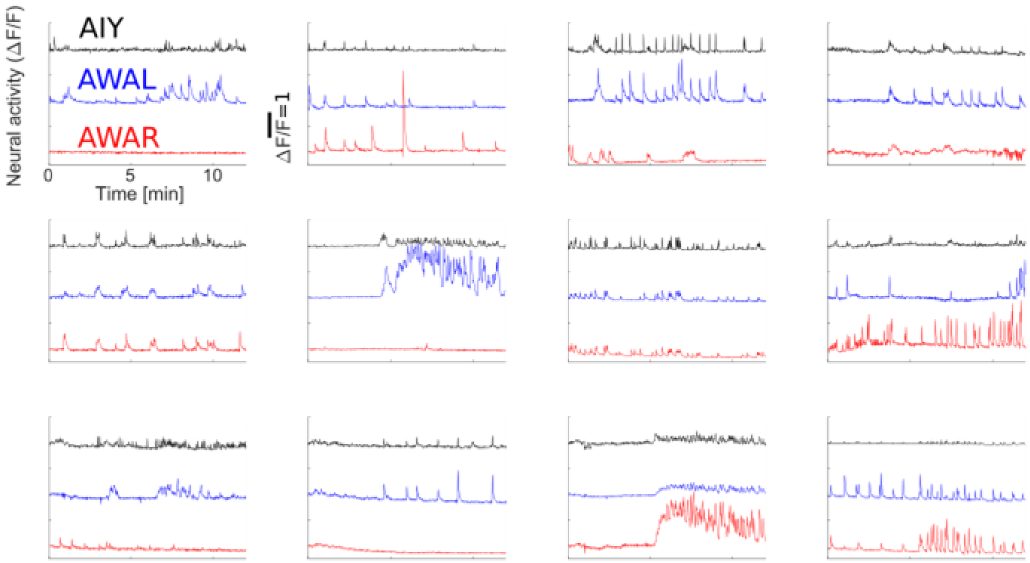

Supplementary figure 10 | Activity of the two bilateral electrically-coupled AWA neurons is not correlated, but their joint activity correlates with the activity of the downstream AIY interneurons. Shown are traces from all 12 animals assayed. AIY (black), AWAR (blue) and AWAL (red). These neural activities are measured in response to a sigmoidal gradient. Notice that the two AWA neurons do not always pulse simultaneously. AIY pulses are shorter compared to AWA pulses ($p<10e-3$), and are positively correlated with the combined activity of AWA neurons ($<\rho>=0.64$, $p<10e-20$).

Supplementary figure 11

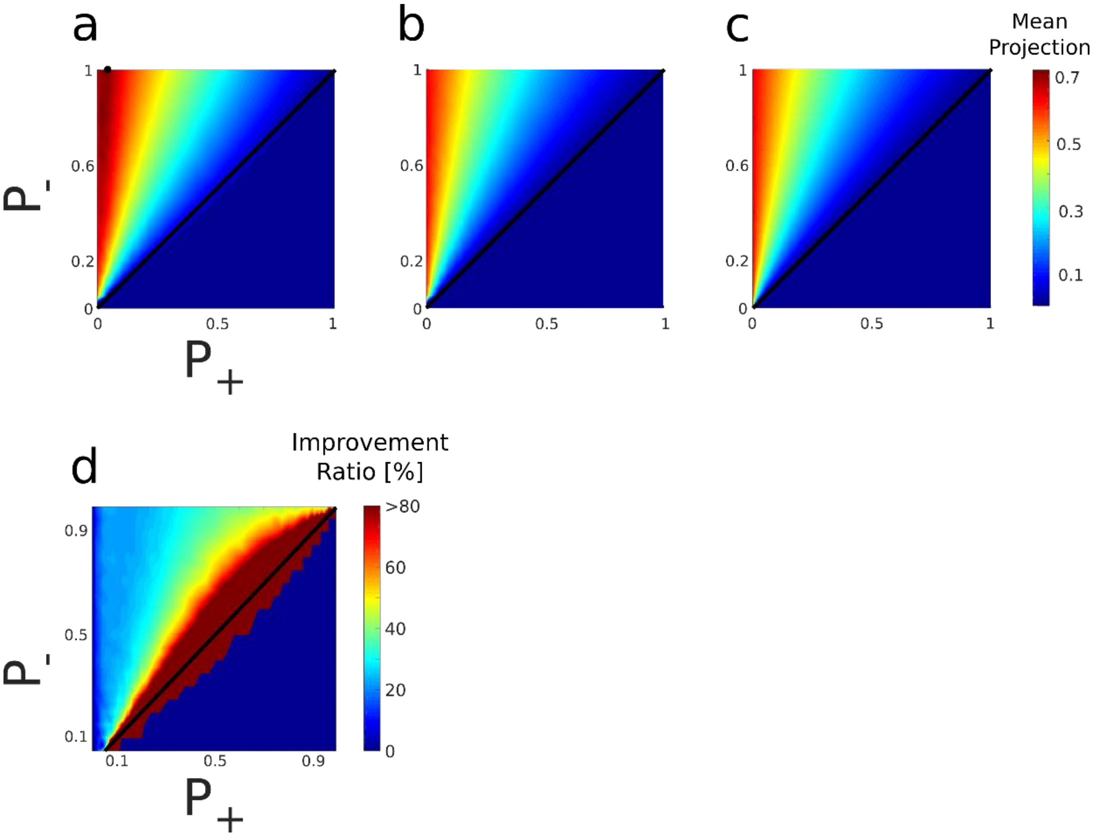

Supplementary figure 11 | The navigation strategy where animals adapt to the first derivative of the gradient is more efficient than the classical biased-random walk strategy. Chemotaxis performance based on simulations in a linear gradient in two strategies: **(a)** with adaptation to the magnitude of the first derivative. **(b)** the classical biased-random walk strategy. Axes denote turning probabilities when experiencing a positive gradient (x-axis, P_+) or a negative gradient (y-axis, P_-). Color marks chemotaxis score as measured by calculating the mean projection of the velocity vector on the optimal trajectory line. Black diagonal line marks the $P_+ = P_-$ line. Black dot in (a) marks the maximal chemotaxis score that can be achieved. Notably, the maximal score is obtained when $P_+ > 0$; this means that when incorporating the first-derivative adaptation strategy, performance is enhanced when animals occasionally reorient (in search of higher slopes) even if continuously sensing an increasing gradient only. **(c)** Results of the analytic solution when obeying the classical biased-random walk strategy in linear gradients. Indeed, (c) and (b) provide identical results. **(d)** The ratio between the two strategies defined as $100 \cdot \frac{first\ der.\ adaptation\ score - classical\ biased\ random\ walk\ score}{classical\ biased\ random\ walk\ score}$. Note that for the entire space where $P_+ > 0$, the strategy of adapting to the first derivative outperforms the classical

biased-random walk strategy. When approaching the $P_+ = P_-$ line (black), this ratio goes to infinity as animals obeying the classical strategy never reach the target.

Supplementary figure 12

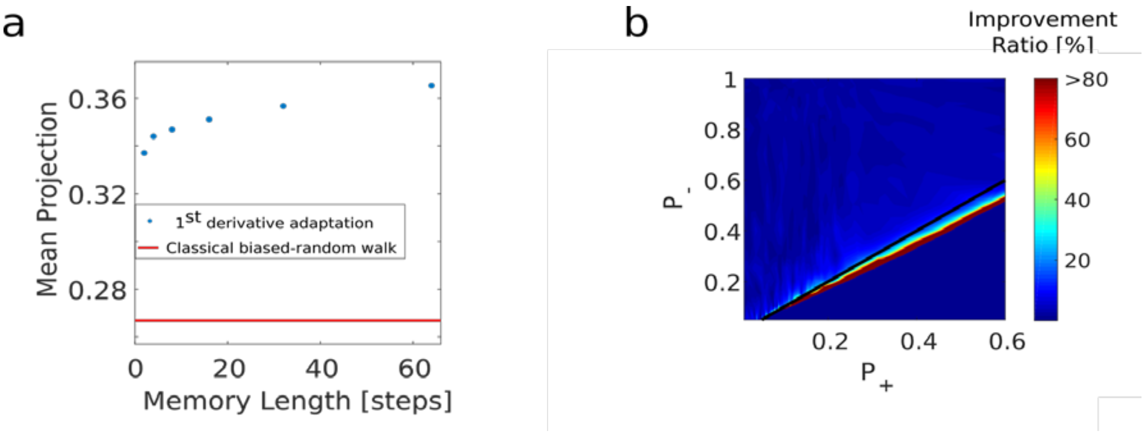

Supplementary figure 12 | Increasing memory length slightly improves chemotaxis performance. We repeated the described simulations in figure 5 for several values of the memory length M. **(a)** The mean projection for increasing lengths of memory as calculated over all values of P_+ and P_- for which at least a single animal was able to reach the target using the classical biased random walk strategy. Red line marks the mean projection calculated for the classical biased random walk strategy. It is evident that increasing the memory length somewhat improves directionality, but even for M=2, a substantial increase over the classical biased random walk strategy is observed. **(b)** Fold-improvement in the performance between M=32 and M=64. Black line marks the $P_+ = P_-$ line. The fold-improvement is defined as $100 \cdot ($ M=64 mean projection- M=32 mean projection)/(M=32 mean projection) For most values of P_+ and P_- fold-improvement is very small, but at the chemotaxis limit, around the black line, fold improvement increases as worms with a shorter memory length can no longer reach the target.

Supplementary figure 13

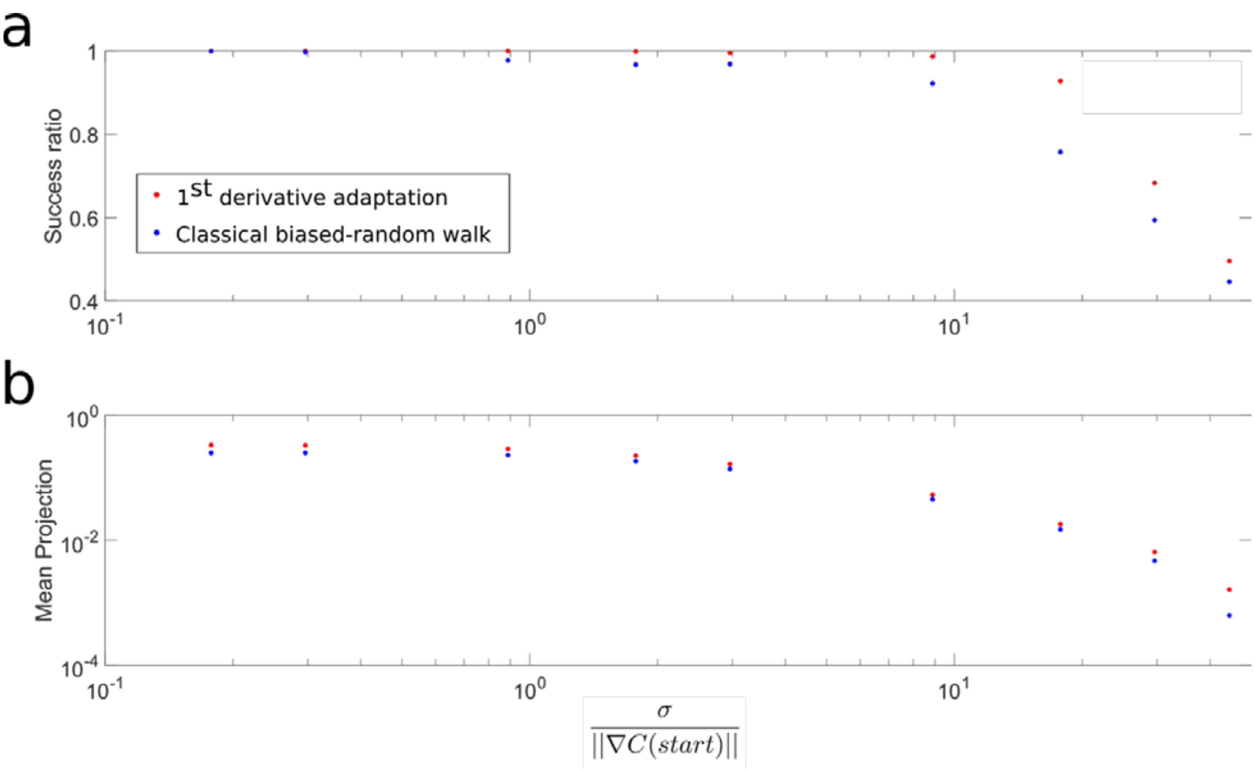

Supplementary figure 13 | In noisy environments, the strategy of adapting to the first derivative outperforms the classical biased random walk strategy. We repeated the simulations (as described for figure 5) for various levels of noise. In each simulation step, noise was drawn from a Gaussian distribution with mean 0 and standard deviation σ. X axis shows the ratio between noise standard deviation and the size of the mathematical gradient at the simulation starting point. **(a)** For each noise level we calculated the success score (SC) for both strategies as the fraction of (P_+,P_-) values for which at least a single simulated animal was able to reach the target. The success ratio is defined as: $successratio = \frac{SC(\sigma)}{SC_{classical\ biased\ random\ walk}(\sigma=0)}$. **(b)** Mean projection was calculated for each strategy and each noise level over all (P_+,P_-) values in which even at the highest noise level simulated at least a single classical biased random walk animal was able to reach the target. For both scores and in all noise levels, the 1st derivative adaptation strategy is superior to the classical biased random walk.

Supplementary figure 14

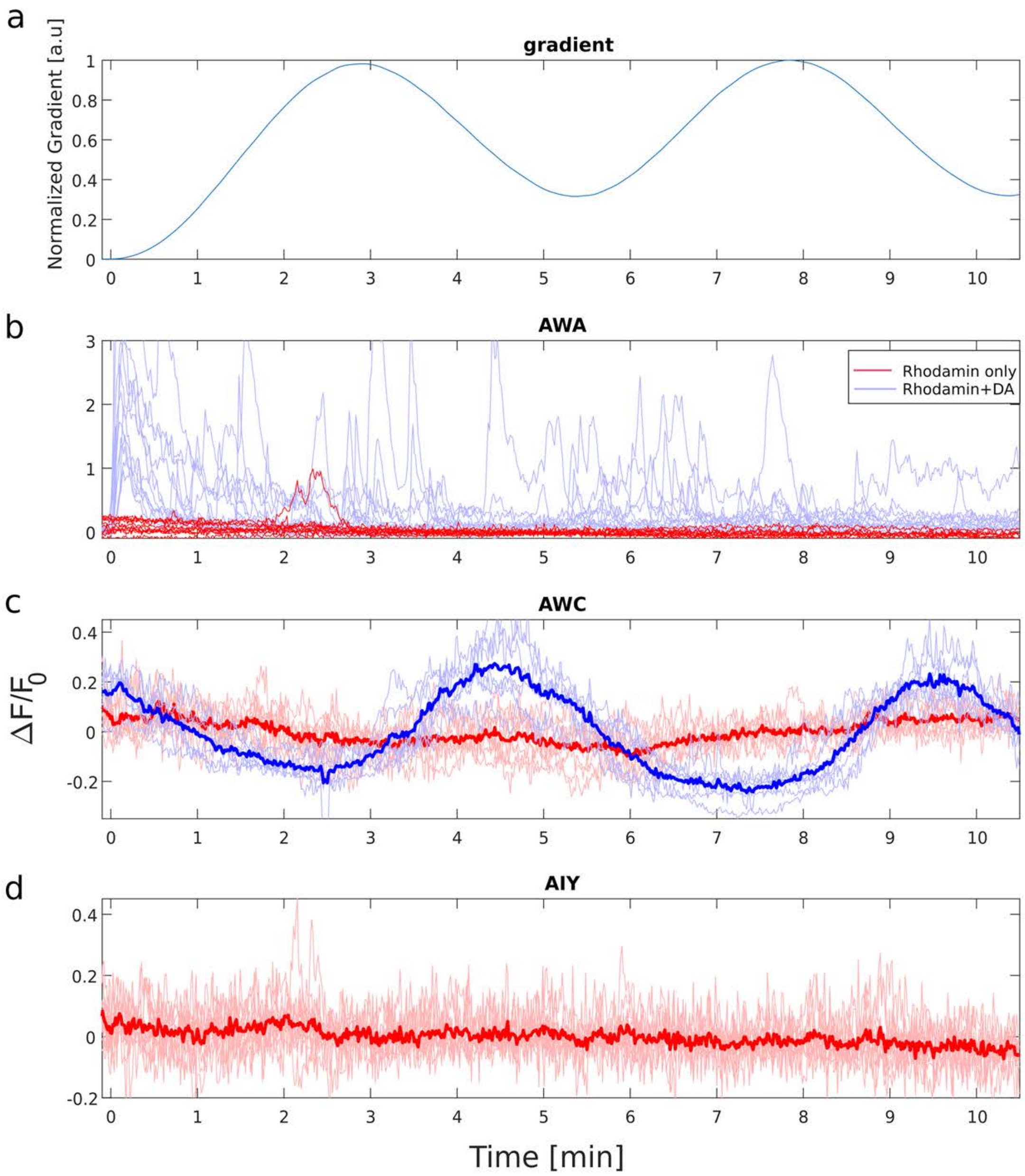

Supplementary figure 14 | Rhodamine, or possible changes in the pressure, do not elicit neural responses, and the observed dynamics are solely in response to diacetyl gradients. We measured neural activity in response to a sinusoidal gradient of rhodamine with and without diacetyl. **(a)** The measured shape of the gradient. Diacetyl concentrations varied between 0.15 µM and 0.8 mM. Rhodamine concentrations varied between 0 and 1 µM. **(b)** Response of AWA neurons to the gradient in (a) with diacetyl (blue). Shown are 12 neural traces

measured from 7 different worms (same experiments as in Fig. 4 a-c). Red, traces to same experimental conditions where the stimulus syringe contained rhodamine only (no diacetyl). Shown are traces for 11 neurons from 7 different worms. **(c)** Response of the AWC^{ON} neuron to the gradient shown in (a) with diacetyl (blue, 7 worms, same experiments as in Fig. 4a-c) and with Rhodamin only (red, 9 worms in total). Bold lines marks the mean fold change activity of these responses. **(d)** Response of AIY neurons to the Rhodamin only gradient (red, 11 worms). Bold red curve is the mean fold change activity. The two small peaks in minutes 2.2 and 2.5 correspond to the two red peaks of AWA shown in (b) as they were recorded from the same animal. Note that when AIY genuinely responds to changes in diacetyl concentrations, it correlates the activity of AWA neurons (Fig. 4f), and typically reaches 100% increase in neural activity (Fig. 4e). Due to the relatively weak neural activity signal in the absence of diacetyl, we subtracted from each of the curves in c and d their linear fit to account for bleaching.

Supplementary figure 15

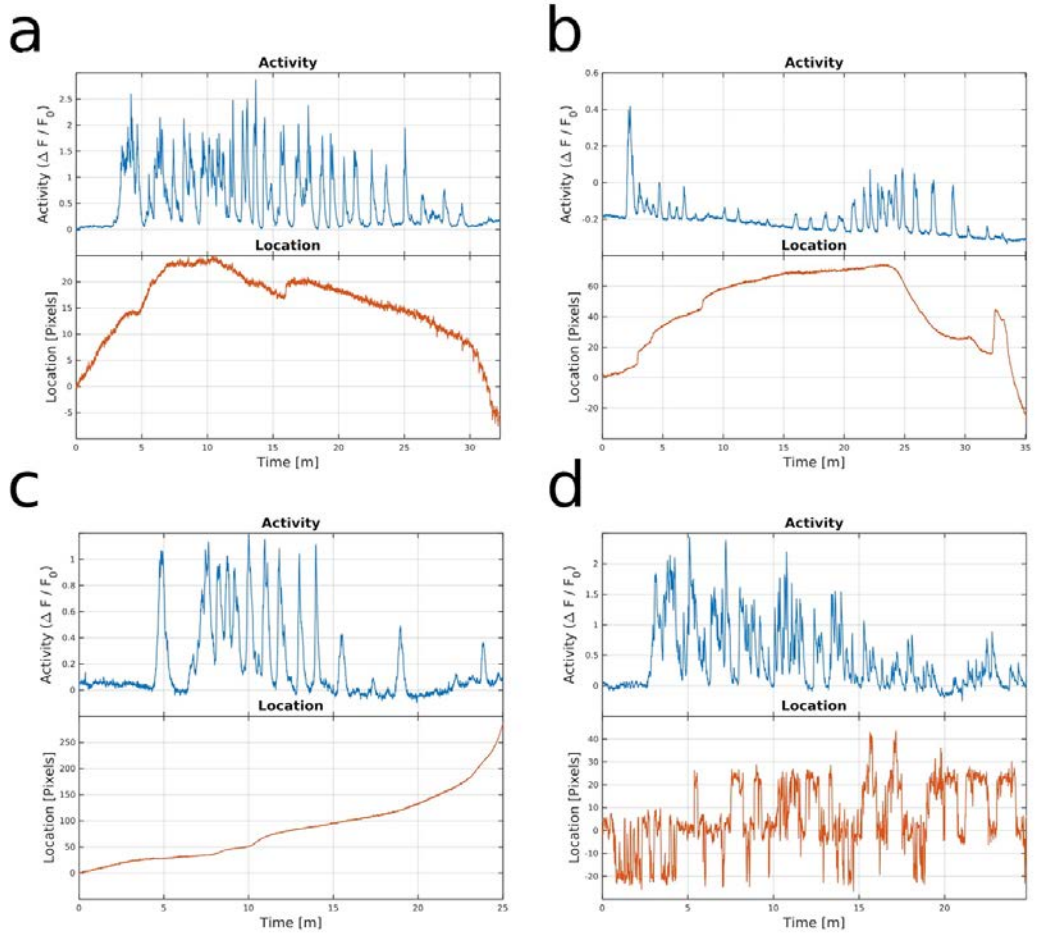

Supplementary figure 15 | Neural activity is not affected by possible movement of the worm within the microfluidic device. (a-d) Plots corresponding to supplementary movies 1 (a), 4(b), 5(c), and 7(d) showing neural activity (top panels, blue), and the corresponding distance of the neuron compared to the beginning of the movie at t=0 (bottom panels, orange). The distance is calculated by the projection of the neuron location on the worm anterior-posterior axis. The mean correlation between the location of the neuron and its activation across n=17 analyzed experiments was not significantly different from 0 ($\bar{\rho} = 0.05$, Wilkoxon Signed Ranksum test, $p = 0.17$). Thus, minute displacements of the worms throughout the experiment do not affect the observed pulsatile responses.

Supplementary figure 16

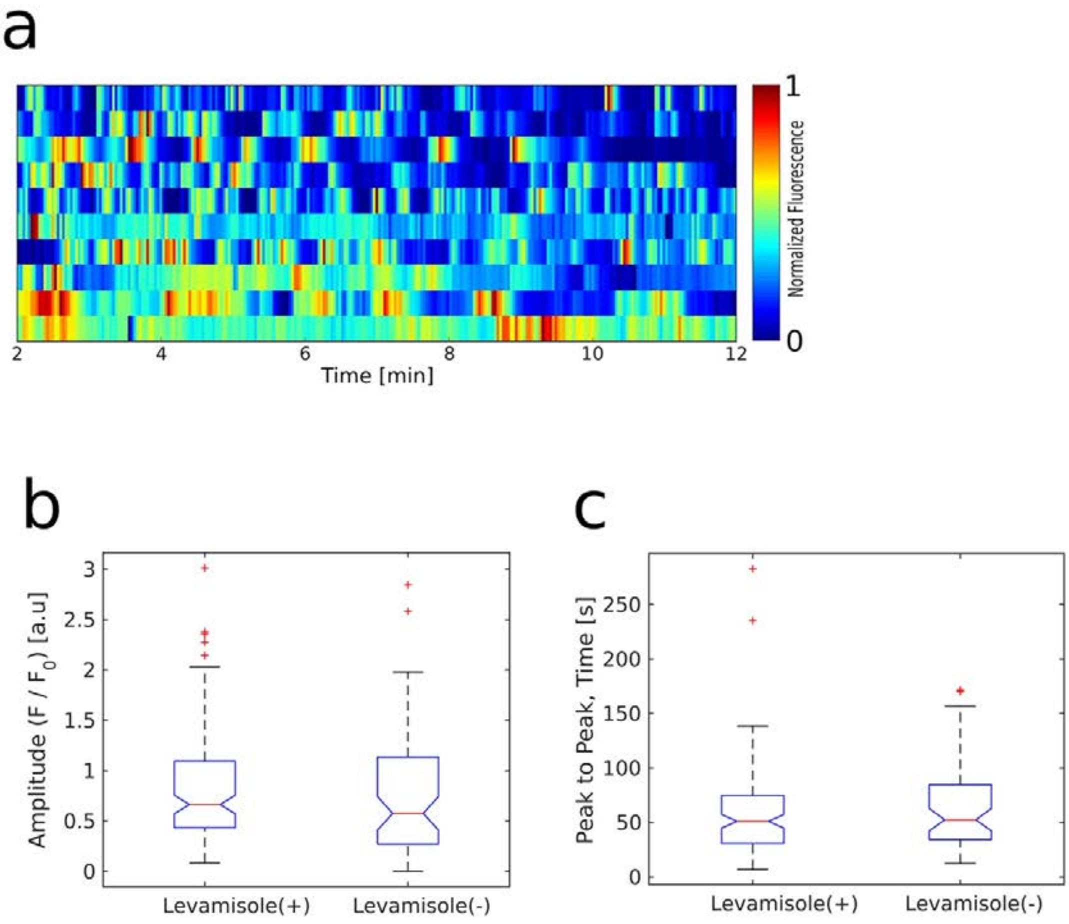

Supplementary figure 16 | Un-anesthetized (awake) worms show similar pulsatile responses as anesthetized worms. (a) A raster plot showing neural activity of un-anesthetized worms presented with a linear gradient (slope = $2\frac{\mu M}{min}$). This is the same gradient used in figure 1e bottom, for anesthetized worms. A comparison of amplitude **(b)** and peak to peak times **(c)** between anesthetized (levamisole+) and un-anesthetized (levamisole-) worms. There are no significant differences in the amplitude, nor in the P2P times, between the two populations (p=0.37 and p=0.23, respectively, Wilcoxon Rank Sum Test). $n_{levamisole(+)}$=123, $n_{levamisole(-)}$=66 pulses, compiled from 7 worms for each condition. The decay time was not compared between the two groups as there were not enough pulses in the group of un-anesthetized worms to which we could fit exponentials. Blue box marks the 0.25 to 0.75 quantiles, and whiskers mark values between the 0.1 and 0.9 quantiles.

Supplementary figure 17

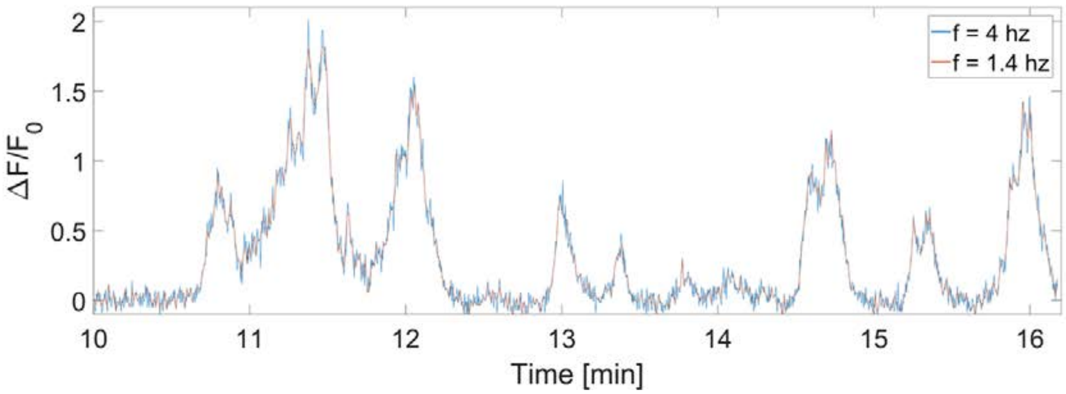

Supplementary figure 17 | Imaging at 1.4 Hz is sufficient to reliably capture the pulsatile activity. AWA neural activity was recorded in response to a diacetyl gradient at a frame rate of 4Hz (blue). The same data was then down-sampled to a frame rate of 1.4 Hz (red). The activity captured with the lower under-sampled frame rate is in high agreement with the one captured with the higher frame rate including all the fine minute peaks. Since the pulses are on timescales of many seconds, measuring at 1.4 Hz faithfully depicts the intricate dynamics so that no activity data is lost, overlooked, or under-sampled due to this lower frame rate.

Supplementary figure 18

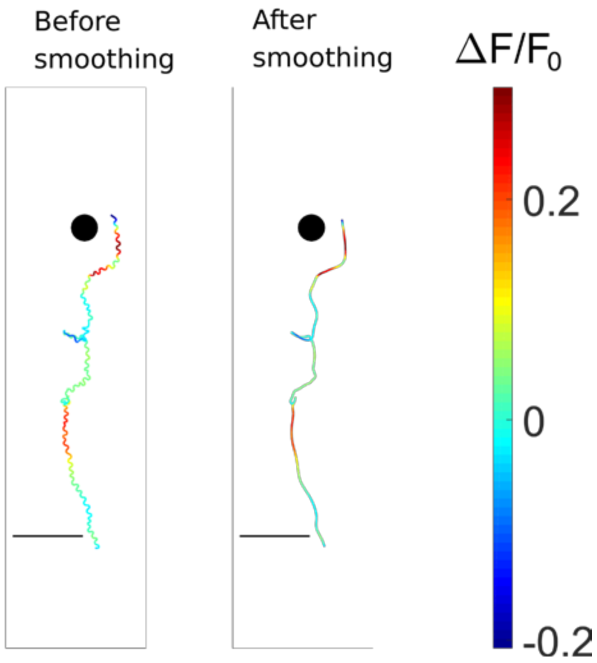

Supplementary figure 18 | The 2D smoothing algorithm smoothens the undulatory tracks but faithfully preserves the navigation trajectories. To accurately analyze deviation of worm trajectory from the target, we applied a 2D smoothing algorithm[6,7]. Shown is the same track as in figure 2b before (left) and after (right) 2D smoothing. The 2D smoothing eliminated the high frequency undulations of the worm movement, but accurately preserved the trajectory. Color indicates AWA fluorescence as measured during chemotaxis. Black scale bar = 5 mm.

Supplementary References

1 Sourjik, V. & Wingreen, N. S. Responding to chemical gradients: bacterial chemotaxis. *Current opinion in cell biology* **24**, 262-268, doi:10.1016/j.ceb.2011.11.008 (2012).

2 Strong, S., Freedman, B., Bialek, W. & Koberle, R. Adaptation and optimal chemotactic strategy for E. coli. *Physical Review E* **57**, 4604 (1998).

3 Itskovits, E., Levine, A., Cohen, E. & Zaslaver, A. A multi-animal tracker for studying complex behaviors. *BMC Biol* **15**, 29, doi:10.1186/s12915-017-0363-9 (2017).

4 Swierczek, N. A., Giles, A. C., Rankin, C. H. & Kerr, R. A. High-throughput behavioral analysis in C. elegans. *Nat Methods* **8**, 592-U112, doi:10.1038/Nmeth.1625 (2011).

5 Larsch, J. *et al.* A Circuit for Gradient Climbing in C. elegans Chemotaxis. *Cell Rep* **12**, 1748-1760, doi:10.1016/j.celrep.2015.08.032 (2015).

6 Garcia, D. A fast all-in-one method for automated post-processing of PIV data. *Experiments in fluids* **50**, 1247-1259, doi:10.1007/s00348-010-0985-y (2011).

7 Garcia, D. Robust smoothing of gridded data in one and higher dimensions with missing values. *Computational statistics & data analysis* **54**, 1167-1178, doi:10.1016/j.csda.2009.09.020 (2010).
