# Supplementary material for: Concerted pulsatile and graded neural dynamics enables efficient chemotaxis in C. elegans
Source: Nat Commun. 2018 Jul 20;9:2866. doi: 10.1038/s41467-018-05151-2 (PMC6054637; doi:10.1038/s41467-018-05151-2)
Supplement: Supplementary file 3 — Description of Additional Supplementary Files [file 41467_2018_5151_MOESM3_ESM.pdf]

Description of Additional Supplementary Files

File Name: Supplementary Movie 1
Description: We measured neural activity (GCaMP signal, green channel, top left) in response to linear gradients of and diacetyl (via rhodamine, red channel, bottom left). From the green channel we extracted the fold change florescence of the neuron (top right, blue) and from the red channel we extracted the measured concentrations (red curve). The measured gradient is indeed in excellent agreement to the predicted gradient (bottom right, black).

File Name: Supplementary Movie 2
Description: Top left – A worm is tracked as it crawls towards a diacetyl source and neural activity of AWA neurons is measure in simultaneously. The tracker registers its location so the trajectory can be extracted (top right). Neural GCaMP fluorescence is at top right (color coded) and a bottom, blue curve. Movie is 9X accelerated.

File Name: Supplementary Movie 3
Description: An exemplary movie depicting the behavioral response of a worm to the light activation of the AWA neuron. While the light is on, and AWA is activated and the worm continues to crawl forward. Once the light switches off, AWA activity decreases, and the worm tends to back and subsequently reorient.

File Name: Supplementary Movie 4
Description: We presented the worm with an exponential diacetyl gradient while simultaneously recording GCaMP signal (green channel, top left) and rhodamine dye concentrations (red channel, bottom left). From the green channel we extract the fold change florescence of the neuron (top right, blue) and from the red channel we extract the measured concentrations (red curve).

File Name: Supplementary Movie 5
Description: We presented the worm with a sigmoidal diacetyl gradient while simultaneously recording GCaMP signal (green channel, top left) and rhodamine dye concentrations (red channel, bottom left). From the green channel we extract the fold change florescence of the neuron (top right, blue) and from the red channel we extract the measured concentrations (red curve).

File Name: Supplementary Movie 6
Description: We imaged the activity of both AWA neurons (middle, bottom) and the sum of the two AIY neurons (top) to a gradient of diacetyl. Although the two AWA neurons are not pulsing in sync, AIY activity seems to correlate both of them.

File Name: Supplementary Movie 7
Description: We presented an un-anesthetized worm with a linear diacetyl gradient while simultaneously recording GCaMP signal (green channel, top left) and rhodamine dye concentrations (red channel, bottom left). From the green channel we extract the fold change florescence of the neuron (top right, blue) and from the red channel we extract the measured concentrations (red curve).
